# The cap‐snatching frequency of a plant bunyavirus from nonsense mRNAs is low but is increased by silencing of *UPF1* or *SMG7*

**DOI:** 10.1111/mpp.13179

**Published:** 2021-12-26

**Authors:** Jing Jin, Yuanyuan She, Ping Qiu, Wenzhong Lin, Wenwen Zhang, Jie Zhang, Zujian Wu, Zhenguo Du

**Affiliations:** ^1^ State Key Laboratory of Ecological Pest Control for Fujian and Taiwan Crops Fuzhou China; ^2^ Plant Virus Research Institute Fujian Agricultural and Forestry University Fuzhou China

**Keywords:** cap‐snatching, nonsense‐mediated decay, plant‐infecting bunyavirus, rice stripe virus, SMG7, UPF1

## Abstract

Bunyaviruses cleave host cellular mRNAs to acquire cap structures for their own mRNAs in a process called cap‐snatching. How bunyaviruses interact with cellular mRNA surveillance pathways such as nonsense‐mediated decay (NMD) during cap‐snatching remains poorly understood, especially in plants. Rice stripe virus (RSV) is a plant bunyavirus threatening rice production in East Asia. Here, with a newly developed system allowing us to present defined mRNAs to RSV in *Nicotiana benthamiana*, we found that the frequency of RSV to target nonsense mRNAs (nsRNAs) during cap‐snatching was much lower than its frequency to target normal mRNAs. The frequency of RSV to target nsRNAs was increased by virus‐induced gene silencing of *UPF1* or *SMG7*, each encoding a protein component involved in early steps of NMD (in an *rdr6* RNAi background). Coincidently, RSV accumulation was increased in the *UPF1*‐ or *SMG7*‐silenced plants. These data indicated that the frequency of RSV to target nsRNAs during cap‐snatching is restricted by NMD. By restricting the frequency of RSV to target nsRNAs, NMD may impose a constraint to the overall cap‐snatching efficiency of RSV. Besides a deeper understanding for the cap‐snatching of RSV, these findings point to a novel role of NMD in plant–bunyavirus interactions.

## Graphical abstract

In contrast to previous assumptions, the cap‐snatching frequency of rice stripe virus, a plant bunyavirus, from nonsense mRNAs is restricted by nonsense‐mediated decay of the host cell.

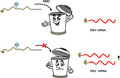

1

Bunyaviruses cleave host cellular mRNAs at a site 10–20 nucleotides (nt) from the cap and use 5′‐terminal cleavage products as primers for transcription of viral genomic RNAs. This mechanism, which results in viral mRNAs with a host‐derived capped RNA leader (CRL) sequence at their 5′ termini, is called cap‐snatching (Olschewski et al., 2020). The major subcellular sites where bunyaviruses perform cap‐snatching remain elusive. Some studies have pointed to cellular processing bodies (PBs; Hopkins et al., 2013, 2015; Ma et al., 2019; Mir et al., 2008; Olschewski et al., 2020). However, PB formation is unnecessary for the infection of some bunyaviruses, suggesting that bunyaviruses also perform cap‐snatching elsewhere (Cheng & Mir, 2012; Olschewski et al., 2020).

Eukaryotic cells occasionally produce nonsense mRNAs (nsRNAs) with premature termination codons (PTCs). These nsRNAs can be translated to truncated proteins that may be deleterious to the cell. Cells use a multistep pathway named nonsense‐mediated decay (NMD) to eliminate nsRNAs (Kervestin & Jacobson, 2012). The subcellular site where each step of NMD takes place remains a matter of debate. However, there is evidence that nsRNAs may be transported to PBs at later stages of NMD (Chang et al., 2007; Kervestin & Jacobson, 2012; Mérai et al., 2013). Besides nsRNAs, NMD regulates many normal transcripts (Drechsel et al., 2013; Lykke‐Andersen & Jensen, 2015). For example, it was estimated that NMD affects more than 20% of the transcriptome in *Arabidopsis* (Raxwal et al., 2020).

Because cap‐snatching and NMD may co‐occur in PBs, nsRNAs (and other NMD‐regulated transcripts) are ideal cap‐snatching targets for bunyaviruses. However, the degradation of nsRNAs may be much faster than that of mRNAs transported to PBs via NMD‐unrelated pathways. This suggests that the chance for a bunyavirus to target nsRNAs is low. The two contradictory predictions are reconciled in a finding of Mir et al. (2008), which showed that a hantavirus seems to have a mechanism to inhibit later stages of NMD: the nucleocapsid protein (NP) of the hantavirus has a cap‐binding activity. By binding to the cap structure of a nsRNA in PBs, it protects the degradation of the nsRNA from the 5′ terminus. With this mechanism, the hantavirus was shown to target a PTC‐containing *GFP* mRNA more than twofold more frequently than a normal *GFP* mRNA during cap‐snatching (Mir et al., 2008). Such a mechanism predicts that deficiencies in early steps of NMD, which reduce nsRNA accumulation in PBs, are detrimental to the hantavirus. This idea was not tested but was supported by a recent report that showed that *Arabidopsis* with a deficiency in an early step of NMD shows increased resistance to a plant bunyavirus named tomato spotted wilt virus (TSWV; Ma et al., 2019). However, whether TSWV frequently targets nsRNAs during cap‐snatching remains unknown.

*Rice stripe tenuivirus* is a species of genus *Tenuivirus* in the family *Phenuiviridae* of the order *Bunyavirales* (Xu et al., 2021). By infecting plants of the Gramineae family, particularly rice, rice stripe virus (RSV) poses a serious threat to crop production in some countries of East Asia (Falk & Tsai, 1998). Although many aspects of RSV have been studied intensively in the past decade (Xu et al., 2021), our understanding of the cap‐snatching of RSV remains poor (Kormelink et al., 2021; Liu et al., 2016, 2018; Yao et al., 2012). We recently showed that RSV can target mRNAs transiently expressed in *Nicotiana benthamiana* using agro‐infiltration (Lin et al., 2020). This provided us with a system to present defined mRNAs to RSV to investigate its cap‐snatching in planta. With the availability of this system and the background described above, we decided to investigate (a) whether RSV targets nsRNAs more frequently in comparison to normal mRNAs during cap‐snatching; (b) how deficiencies in early steps of NMD influence the cap‐snatching of RSV from nsRNAs; and (c) how deficiencies in early steps of NMD influence the infection of RSV.

A competition assay was used to investigate whether RSV targets nsRNAs more frequently in comparison to normal mRNAs. In this assay, *Agrobacterium tumefaciens* cell cultures carrying the plasmid pCHF_3_‐C^12^, which expresses a normal green fluorescent protein gene (*GFP*) mRNA (GFP‐n), or pCHF_3_‐C^11^‐PTC, which expresses a mutant *GFP* mRNA with a PTC at codon 3 (GFP‐m), were mixed at a ratio of 1:1 (see File S1 for experimental procedures). The mixture was infiltrated into a leaf of RSV‐infected *N. benthamiana* (Figure 1a). The infiltrated leaf patch was collected at 3 days post‐agro‐infiltration (dpi) and RSV *NP* mRNA in the collected leaf patch was deep sequenced with a method briefly indicated in Figure 1b. The *NP* mRNA sequences are highly heterogenous with respect to their CRLs because RSV targets a great diversity of cellular mRNAs during cap‐snatching (Lin et al., 2017; Liu et al., 2018). For simplicity, RSV *NP* mRNA sequences with their CRLs acquired from GFP‐m and GFP‐n are called GFP‐m‐NP and GFP‐n‐NP, respectively. GFP‐m‐NP and GFP‐n‐NP can be distinguished from each other because RSV cleaves GFP‐m and GFP‐n at C_12_ and C_11_ (Figure 1a), respectively, acquiring two CRLs differing in length (Lin et al., 2020).

**FIGURE 1:**
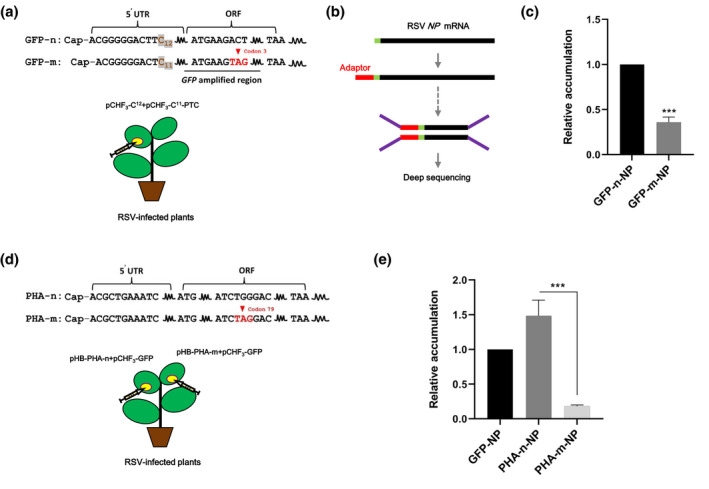
The low frequency of RSV to target nonsense mRNAs during cap‐snatching. (a) A diagrammatic sketch of GFP‐n and GFP‐m and the way by which they were expressed in RSV‐infected *Nicotiana benthamiana*. The *GFP* amplified region (underlined) was used to evaluate the relative accumulation of GFP‐n and GFP‐m by deep sequencing. (b) Deep sequencing of RSV *NP* mRNA. The experiment was performed as described previously (Lin et al., 2017; Liu et al., 2018). Briefly, the *NP* mRNA was ligated to an adaptor after decapping (with total RNA as the starting material). The oligo‐tagged *NP* mRNA was deep sequenced after reverse transcription‐PCR and library construction. The green‐coloured region of the mRNA (in black) indicates capped RNA leader. The red‐coloured fragment indicates the adaptor ligated to the mRNA. The purple‐coloured region indicates the adaptor ligated to the PCR amplicon during library construction. (c) The relative accumulation of GFP‐m‐NP to GFP‐n‐NP. The value of GFP‐n‐NP was set to be 1. (d) A diagrammatic sketch of PHA‐n and PHA‐m and the way by which they were expressed in RSV‐infected *N*. *benthamiana*. (e) The relative accumulation of PHA‐n‐NP to GFP‐NP and PHA‐m‐NP to GFP‐NP. The value of GFP‐NP was set to be 1. Each value is the mean (±*SEM*) of three biological replicates. Different letters indicate statistically significant differences as determined by one‐way analysis of variance with Tukey's post hoc test (*p* < 0.05)

The assay was done in triplicate. The total number of *NP* mRNA sequences obtained from each replicate was 80,664, 99,962, and 79,889. The numbers of GFP‐m‐NP in the three replicates were 319, 148, and 194, whereas those of GFP‐n‐NP were 677, 466, and 662. Thus, the accumulation of GFP‐m‐NP relative to that of GFP‐n‐NP (GFP‐m‐NP/GFP‐n‐NP) in each replicate deviated slightly from a mean value of 0.36:1 (Figure 1c). This indicated that GFP‐m had been targeted nearly threefold less frequently than GFP‐n by the cap‐snatching of RSV.

As the CRL donated by GFP‐n is one nucleotide longer than that donated by GFP‐m, this observation can be explained by a preference of RSV for longer CRLs. A reciprocal experiment was done to rule out this possibility. In this experiment, GFP‐n donated an 11‐nt CRL, whereas GFP‐m donated a 12‐nt CRL to RSV. The same result was obtained: GFP‐m was much less frequently targeted than was GFP‐n (see File S2 for the raw data).

A bean *phytohemagglutinin* (PHA) mRNA containing a PTC at codon 79 (PHA‐m), which has been used as a nsRNA by researchers dissecting NMD of plants, was used to confirm the low frequency of RSV to target nsRNAs (Kertesz et al., 2006). PHA‐m was expressed using the binary vector pHB. PHA‐n, the normal PHA mRNA, was expressed with the same vector. Because the 5′‐terminal sequences of PHA‐m and PHA‐n are identical (Figure 1d), a direct competition assay like that carried out for GFP‐m and GFP‐n is infeasible. As an alternative, pHB‐PHA‐m and pHB‐PHA‐n were each agro‐infiltrated independently into a different leaf of the same plant. *A. tumefaciens* cell cultures carrying pCHF_3_, which expresses a *GFP* mRNA, were included at a ratio of 1:1 in each agro‐infiltration (Figure 1d). In this way, we used two independent competition assays, one between PHA‐m and *GFP* and the other between PHA‐n and *GFP*, to investigate whether RSV targets PHA‐m more frequently in comparison to PHA‐n. Sample collection and RSV *NP* deep sequencing were performed as done above. Similarly, RSV *NP* mRNA sequences with CRLs derived from PHA‐m, PHA‐n, and *GFP* were called PHA‐m‐NP, PHA‐n‐NP, and GFP‐NP, respectively.

The accumulation of PHA‐m‐NP relative to GFP‐NP (PHA‐m‐NP/GFP‐NP) was compared to that of PHA‐n‐NP relative to GFP‐NP (PHA‐n‐NP/GFP‐NP) (File S2). As shown in Figure 1e, the mean PHA‐m‐NP/GFP‐NP value was about 0.18:1. In contrast, PHA‐n‐NP/GFP‐NP had a mean value of 1.49:1. Thus, like GFP‐m, PHA‐m was targeted much less frequently than normal mRNAs by the cap‐snatching of RSV.

To investigate how deficiencies in early steps of NMD influence the cap‐snatching of RSV from nsRNAs, the competition assays described above were carried out in *N. benthamiana* whose *UPF1* or *SMG7*, each encoding a protein component involved in early steps of NMD, had been silenced using virus‐induced gene silencing (VIGS). To do this, a cDNA fragment of *UPF1* or *SMG7* was cloned into the tobacco rattle virus (TRV)‐based VIGS vector pTRV2 (Liu et al., 2002). *A. tumefaciens* cell cultures containing pTRV2‐UPF1, pTRV2‐SMG7, or pTRV2 carrying a fragment of *luciferase* (pTRV2‐LUC), which was used as a control, were each mixed with cultures containing pTRV1 before being infiltrated to leaves of *N. benthamiana*. At 10 dpi, when *UPF1* and *SMG7* had been silenced by about 68% and 76% (data not shown), respectively, the *N. benthamiana* was rub‐inoculated with RSV. Twenty days after the rub‐inoculation, agro‐infiltration, sample collection, and deep sequencing of RSV *NP* mRNA were performed as done above. Because reducing *UPF1* or *SMG7* expression may enhance the activity of RDR6‐mediated gene silencing, which may influence data interpretation, all these assays were done with an *rdr6* RNAi line of *N. benthamiana* (Liu & Chen, 2016; Moreno et al., 2013; Qu et al., 2005).

Before deep sequencing of RSV *NP* mRNAs, we investigated how the relative accumulation of GFP‐m or PHA‐m was influenced in *UPF1*‐ or *SMG7*‐silenced plants. The accumulation of PHA‐m relative to that of PHA‐n was detected with reverse transcription‐quantitative PCR (RT‐qPCR). As shown in Figure 2a, the relative accumulation of PHA‐m was increased 2.2‐fold in *UPF1*‐silenced plants in comparison to control plants (plants preinfected by TRV‐LUC). In contrast, its relative accumulation was unchanged in *SMG7*‐silenced plants. The relative accumulation of GFP‐m and GFP‐n was studied with a different approach. In this approach, total RNA extracted from leaf patches co‐expressing GFP‐m and GFP‐n was reverse transcribed using a random primer. The cDNA was PCR amplified using a primer pair (corresponding to a region underlined in Figure 1a) flanking codon 3 of GFP‐m/GFP‐n and the PCR amplicon was deep sequenced. Because GFP‐m and GFP‐n differ by one nucleotide at codon 3, the RT‐PCR sequences corresponding to the two mRNA molecules can be distinguished from each other. As shown in Figure 2b, this experiment showed that the accumulation of GFP‐m was increased 2.63‐fold in *SMG7*‐silenced plants but was unchanged in *UPF1*‐silenced plants.

**FIGURE 2:**
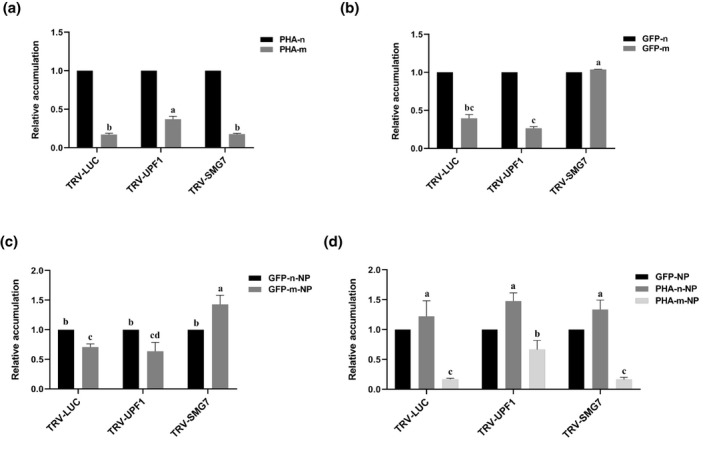
The increased frequency of RSV to target nonsense RNAs in *UPF1‐* or *SMG7*‐silenced *Nicotiana benthamiana* (in an *rdr6* RNAi background). (a) The relative accumulation of PHA‐m in each type of plant. (b) The relative accumulation of GFP‐m in each type of plant. (c) The relative accumulation of GFP‐m‐NP to GFP‐n‐NP in each type of plant. (d) The relative accumulation of PHA‐n‐NP to GFP‐NP or PHA‐m‐NP to GFP‐NP in each type of plant. Different letters indicate statistically significant differences as determined by one‐way analysis of variance with Tukey's post hoc test (*p* < 0.05)

The observation that silencing of *UPF1* or *SMG7* each influenced the relative accumulation of only one nsRNA is unexpected. However, this is consistent with a recent report showing that UPF1 or SMG7 may each regulate an overlapping but different set of cellular transcripts (Raxwal et al., 2020). Alternatively, the residual *UPF1/SMG7* after VIGS may be still enough to commit GFP‐m/PHA‐m to NMD. Whatever the possibility, this offered us a unique opportunity to see whether the effects of *UPF1*/*SMG7* on the frequency by which RSV targets GFP‐m/PHA‐m correlate with their effects on the accumulation of the two nsRNAs.

The values of GFP‐m‐NP/GFP‐n‐NP are presented in Figure 2c (raw data in File S2). The mean GFP‐m‐NP/GFP‐n‐NP value in *UPF1*‐silenced plants was comparable to that in control plants. In contrast, the mean GFP‐m‐NP/GFP‐n‐NP value in *SMG7*‐silenced plants was about twofold higher than that in control plants. Notably, the number of GFP‐m‐NP is larger than that of GFP‐n‐NP in data sets obtained from *SMG7*‐silenced plants. The values of PHA‐m‐NP/GFP‐NP and PHA‐n‐NP/GFP‐NP are presented in Figure 2d (raw data in File S2). As shown, PHA‐n‐NP/GFP‐NP values in all plants are comparable to each other. PHA‐m‐NP/GFP‐NP values in *SMG7*‐silenced plants were comparable to those in control plants, indicating that silencing of *SMG7* did not affect the frequency by which RSV targets PHA‐m. However, the mean PHA‐m‐NP/GFP‐NP value was increased about fourfold in *UPF1*‐silenced plants relative to those in control plants.

These data indicated that the effects of *UPF1*/*SMG7* on the frequency by which RSV targets GFP‐m/PHA‐m correlate well with their effects on the accumulation of the two nsRNAs. To put it in other words, the frequency of RSV to target a nsRNA is increased in plants that had lost the ability to commit that nsRNA to NMD.

Typically, RSV causes mosaic, yellowing, and curling of upper leaves in *N. benthamiana*. These symptoms seemed to be milder in *UPF1*‐ or *SMG7*‐silenced plants than in control plants (Figure 3a). However, *UPF1*‐ or *SMG7*‐silenced plants showed a decrease in their width (Figure 3b). This symptom was not observed in control plants, nor in *UPF1*‐ or *SMG7*‐silenced plants that had not been infected with RSV (Figure 3b; data not shown). To investigate how silencing of *UPF1* or *SMG7* had influenced the susceptibility of *N. benthamiana* to RSV, the accumulation of the virion‐sense and complementary‐sense RSV RNA3 (vRNA3 and vcRNA3) in *UPF1*‐ or *SMG7*‐silenced plants was detected using a strand‐specific RT‐qPCR adapted from Kawakami et al. (2011). Silencing of *SMG7* significantly increased the accumulation of vcRNA3. The accumulation of vRNA3 was also discernibly increased, although this was not supported by statistical analysis. VIGS of *UPF1*, on the other hand, significantly increased the accumulation of both vRNA3 and vcRNA3 (Figure 3c,d). Northern blotting with a biotin‐labelled probe that detects both vRNA3 and vcRNA3 was used to substantiate the results of RT‐qPCR. Considering the great variation of RSV accumulation in each plant, each sample contained pooled upper leaves of six to eight different plants in northern blotting. As shown in Figure 3e, the average accumulation level of RSV RNA3 was much higher in *UPF1*‐ or *SMG7*‐silenced than in control plants. Altogether, these data indicated that silencing of either *UPF1* or *SMG7* increased the susceptibility of *N. benthamiana* to RSV.

**FIGURE 3:**
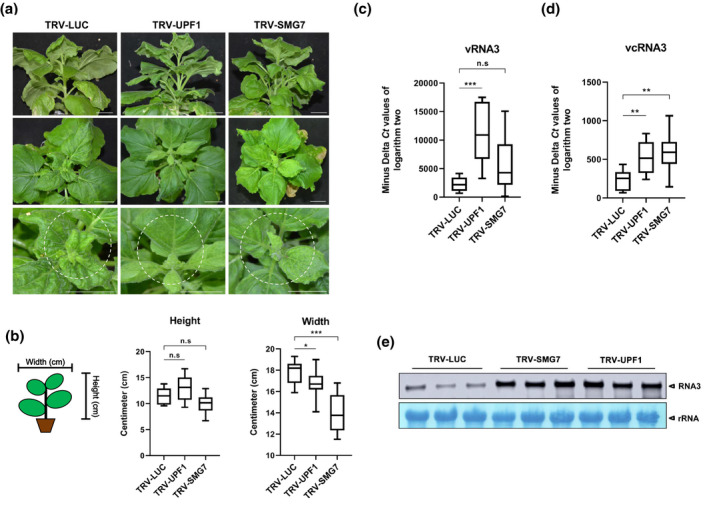
The increased RSV accumulation in *UPF1*‐ or *SMG7*‐silenced *Nicotiana benthamiana* (in an *rdr6* RNAi background). (a) The phenotypes of *LUC*‐, *SMG7*‐, or *UPF*‐silenced *N. benthamiana* 20 days after RSV infection. Scale bar =2 cm. (b) The width and height of *LUC*‐, *SMG7*‐, or *UPF*‐silenced *N. benthamiana* 20 days after RSV infection. Statistical significance was calculated using Student's *t* test (***p < 0.001, *p < 0.05, n.s *p* > 0.05). (c) and (d) The relative accumulation of RSV vRNA3 or vcRNA3. The 2^−Δ*C*t(target, reference)^ values, determined using a strand‐specific reverse transcription quantitative PCR, of 8–10 samples were plotted on the box for each type of plant. Statistical significance was calculated using Student's *t* test (***p < 0.001, **p < 0.01, n.s *p* > 0.05). (e) Northern blot to detect the accumulation of RSV RNA3 in *LUC*‐, *UPF1*‐, or *SMG7*‐silenced plants at 20 days after RSV rub‐inoculation. rRNA was used as the loading control

In all, by using a recently established system that allows us to artificially present defined mRNAs to RSV, this study for the first time investigated the cap‐snatching of a plant bunyavirus from nsRNAs. In contrast to a previous report for a hantavirus (Mir et al., 2008), RSV targets nsRNAs much less frequently than it targets normal mRNAs. The frequency of RSV to target nsRNAs was increased in *UPF1*‐ or *SMG7*‐silenced plants, indicating that NMD is responsible for the low frequency of RSV to target nsRNAs.

Assuming that nsRNAs are transported to PBs at later stages of NMD in plants, our findings can be interpreted in two different ways. First, RSV performs cap‐snatching mainly in the diffuse cytoplasm. Secondly, RSV performs cap‐snatching in PBs but lacks a mechanism to cope with the high rate of nsRNA degradation in PBs. Given that diverse bunyaviruses including one belonging to the same family as RSV have been suggested to use PBs as important sites for cap‐snatching (Hopkins et al., 2013), we hypothesize that the second explanation is likely to be correct. However, our data suggest that PBs are not the sole sites for the cap‐snatching of RSV, otherwise it will be difficult to explain the increased frequency of RSV to target nsRNAs in *UPF1*‐ or *SMG7*‐silenced plants. Is it possible that neither of GFP‐m and PHA‐m goes to PBs at later stages of NMD? We cannot rule out this possibility. If this is true, the interpretation of our data becomes a little more complex. However, our conclusion that NMD restricts the frequency of RSV to target nsRNAs seems to be inarguable.

Given the complex interactions between viruses and NMD of their host cells, the mechanisms underlying the increased accumulation of RSV in *UPF1*‐ or *SMG7*‐silenced *N. benthamiana* are uncertain at present (Balistreri et al., 2017; Li & Wang, 2019). A plausible explanation, however, is that nsRNAs as well as other transcripts regulated by NMD were accumulated in these plants. This leads to a larger mRNA pool that is available for RSV to perform cap‐snatching. If this explanation is true, our finding points to a novel role of NMD in plant–bunyavirus interactions, that is, NMD may limit the infection of bunyaviruses by posing a constraint to their cap‐snatching.

## CONFLICT OF INTEREST

The authors declare that they have no conflict of interest.

## Supporting information


**FILE S1.** Materials and methods; Table S1 Primer sequences

**FILE S2.** Table S2 Statistics for data sets obtained from wild‐type *Nicotiana benthamiana* co‐expressing GFP‐m and GFP‐n (the reciprocal experiment); Table S3 Statistics for data sets obtained from wild‐type *N. benthamiana* co‐expressing PHA‐m/PHA‐n and GFP; Table S4 Statistics for data sets obtained from *UPF1*‐silenced, *SMG7*‐silenced, or control *N. benthamiana* co‐expressing GFP‐m and GFP‐n; Table S5 Statistics for data sets obtained from *UPF1*‐silenced, *SMG7*‐silenced, or control *N. benthamiana* co‐expressing PHA‐m/PHA‐n and GFP

## Data Availability

The data that support the findings of this study are available from the corresponding author upon reasonable request.

## References

[mpp13179-bib-0001] Balistreri, G. , Bognanni, C. & Mhlemann, O. (2017) Virus escape and manipulation of cellular nonsense‐mediated mRNA decay. Viruses, 9, 24. 10.3390/v9010024 PMC5294993 28124995

[mpp13179-bib-0002] Chang, Y.F. , Imam, J.S. & Wilkinson, M.F. (2007) The nonsense‐mediated decay RNA surveillance pathway. Annual Review of Biochemistry, 76, 51–74. 10.1146/annurev.biochem.76.050106.093909 17352659

[mpp13179-bib-0003] Cheng, E. & Mir, M.A. (2012) Signatures of host mRNA 5′ terminus for efficient hantavirus cap snatching. Journal of Virology, 86, 10173–10185. 22787213 10.1128/JVI.05560-11 PMC3446632

[mpp13179-bib-0004] Drechsel, G. , Kahles, A. , Kesarwani, A.K. , Stauffer, E. , Behr, J. , Drewe, P. et al. (2013) Nonsense‐mediated decay of alternative precursor mRNA splicing variants is a major determinant of the *Arabidopsis* steady state transcriptome. The Plant Cell, 25, 3726–3742. 24163313 10.1105/tpc.113.115485 PMC3877825

[mpp13179-bib-0005] Falk, B.W. & Tsai, J.H. (1998) Biology and molecular biology of viruses in the genus *Tenuivirus* . Annual Review of Phytopathology, 36, 139–163. 10.1146/annurev.phyto.36.1.139 15012496

[mpp13179-bib-0006] Hopkins, K.C. , McLane, L.M. , Maqbool, T. , Panda, D. , Gordesky‐Gold, B. & Cherry, S. (2013) A genome‐wide RNAi screen reveals that mRNA decapping restricts bunyaviral replication by limiting the pools of Dcp2‐accessible targets for cap‐snatching. Genes and Development, 27, 1511–1525. 23824541 10.1101/gad.215384.113 PMC3713431

[mpp13179-bib-0007] Hopkins, K.C. , Tartell, M.A. , Herrmann, C. , Hackett, B.A. , Taschuk, F. , Panda, D. et al. (2015) Virus‐induced translational arrest through 4EBP1/2‐dependent decay of 5′‐TOP mRNAs restricts viral infection. Proceedings of the National Academy of Sciences of the United States of America, 112, E2920–E2929. 26038567 10.1073/pnas.1418805112 PMC4460451

[mpp13179-bib-0008] Kawakami, E. , Watanabe, T. , Fujii, K. , Goto, H. , Watanabe, S. , Noda, T. et al. (2011) Strand‐specific real‐time RT‐PCR for distinguishing influenza vRNA, cRNA, and mRNA. Journal of Virological Methods, 173, 1–6. 21185869 10.1016/j.jviromet.2010.12.014 PMC3049850

[mpp13179-bib-0009] Kertész, S. , Kerényi, Z. , Mérai, Z. , Bartos, I. , Pálfy, T. , Barta, E. et al. (2006) Both introns and long 3′‐UTRs operate as cis‐acting elements to trigger nonsense‐mediated decay in plants. Nucleic Acids Research, 34, 6147–6157. 17088291 10.1093/nar/gkl737 PMC1693880

[mpp13179-bib-0010] Kervestin, S. & Jacobson, A. (2012) NMD: a multifaceted response to premature translational termination. Nature Reviews Molecular Cell Biology, 13, 700–712. 23072888 10.1038/nrm3454 PMC3970730

[mpp13179-bib-0011] Kormelink, R. , Verchot, J. , Tao, X. & Desbiez, C. (2021) The Bunyavirales: the plant‐infecting counterparts. Viruses, 13, 842. 34066457 10.3390/v13050842 PMC8148189

[mpp13179-bib-0012] Li, F.F. & Wang, A.M. (2019) RNA‐targeted antiviral immunity: more than just RNA silencing. Trends in Microbiology, 27, 792–805. 31213342 10.1016/j.tim.2019.05.007

[mpp13179-bib-0013] Lin, W. , Qiu, P. , Jin, J. , Liu, S. , Ul Islam, S. , Yang, J. et al. (2017) The cap snatching of segmented negative sense RNA viruses as a tool to map the transcription start sites of heterologous co‐infecting viruses. Frontiers in Microbiology, 8, 2519. 29312219 10.3389/fmicb.2017.02519 PMC5735111

[mpp13179-bib-0014] Lin, W. , Wu, R. , Qiu, P. , Jing, J. , Yang, Y. , Wang, J. et al. (2020) A convenient in vivo cap donor delivery system to investigate the cap snatching of plant bunyaviruses. Virology, 539, 114–120. 31710910 10.1016/j.virol.2019.10.017

[mpp13179-bib-0015] Liu, L. & Chen, X.M. (2016) RNA quality control as a key to suppressing RNA silencing of endogenous genes in plants. Molecular Plant, 9, 826–836. 27045817 10.1016/j.molp.2016.03.011 PMC5123867

[mpp13179-bib-0016] Liu, X. , Jin, J. , Qiu, P. , Gao, F. , Lin, W. , Xie, G. et al. (2018) Rice stripe tenuivirus has a greater tendency to use the prime‐and‐realign mechanism in transcription of genomic than in transcription of antigenomic template RNAs. Journal of Virology, 92, e01414‐17. 29046442 10.1128/JVI.01414-17 PMC5730792

[mpp13179-bib-0017] Liu, X. , Xiong, G. , Qiu, P. , Du, Z. , Kormelink, R. , Zheng, L. et al. (2016) Inherent properties not conserved in other tenuiviruses increase priming and realignment cycles during transcription of *Rice stripe virus* . Virology, 496, 287–298. 27393974 10.1016/j.virol.2016.06.018

[mpp13179-bib-0018] Liu, Y. , Schiff, M. , Marathe, R. & Dinesh‐Kumar, S.P. (2002) Tobacco *Rar1*, *EDS1* and *NPR1*/*NIM1* like genes are required for *N*‐mediated resistance to tobacco mosaic virus. The Plant Journal, 30, 415–429. 12028572 10.1046/j.1365-313x.2002.01297.x

[mpp13179-bib-0019] Lykke‐Andersen, S. & Jensen, T.H. (2015) Nonsense‐mediated mRNA decay: an intricate machinery that shapes transcriptomes. Nature Reviews Molecular Cell Biology, 16, 665–677. 26397022 10.1038/nrm4063

[mpp13179-bib-0020] Ma, X.F. , Zhou, Y.J. & Moffett, P. (2019) Alterations in cellular RNA decapping dynamics affect tomato spotted wilt virus cap snatching and infection in *Arabidopsis* . New Phytologist, 224, 789–803. 10.1111/nph.16049 31292958

[mpp13179-bib-0021] Mérai, Z. , Benkovics, A.H. , Nyikó, T. , Debreczeny, M. , Hiripi, L. , Kerényi, Z. et al. (2013) The late steps of plant nonsense‐mediated mRNA decay. The Plant Journal, 73, 50–62. 22974464 10.1111/tpj.12015

[mpp13179-bib-0022] Mir, M.A. , Duran, W.A. , Hjelle, B.L. , Ye, C. & Panganiban, A.T. (2008) Storage of cellular 5′ mRNA caps in P bodies for viral cap‐snatching. Proceedings of the National Academy of Sciences of the United States of America, 105, 19294–19299. 19047634 10.1073/pnas.0807211105 PMC2614755

[mpp13179-bib-0023] Moreno, A.B. , Martínez de Alba, A.E. , Bardou, F. , Crespi, M.D. , Vaucheret, H. , Maizel, A. et al. (2013) Cytoplasmic and nuclear quality control and turnover of single‐stranded RNA modulate post‐transcriptional gene silencing in plants. Nucleic Acids Research, 41, 4699–4708. 23482394 10.1093/nar/gkt152 PMC3632135

[mpp13179-bib-0024] Olschewski, S. , Cusack, S. & Rosenthal, M. (2020) The cap‐snatching mechanism of bunyaviruses. Trends in Microbiology, 28, 293–303. 31948728 10.1016/j.tim.2019.12.006

[mpp13179-bib-0025] Qu, F. , Ye, X.H. , Hou, G.C. , Sato, S. , Clemente, T.E. & Morris, T.J. (2005) RDR6 has a broad‐spectrum but temperature‐dependent antiviral defense role in *Nicotiana benthamiana* . Journal of Virology, 79, 15209–15217. 16306592 10.1128/JVI.79.24.15209-15217.2005 PMC1316014

[mpp13179-bib-0026] Raxwal, V.K. , Simpson, C.G. , Gloggnitzer, J. , Entinze, J.C. , Guo, W. , Zhang, R. et al. (2020) Nonsense‐mediated RNA decay factor UPF1 is critical for posttranscriptional and translational gene regulation in *Arabidopsis* . The Plant Cell, 32, 2725–2741. 32665305 10.1105/tpc.20.00244 PMC7474300

[mpp13179-bib-0027] Xu, Y. , Fu, S. , Tao, X. & Zhou, X. (2021) Rice stripe virus: exploring molecular weapons in the arsenal of a negative‐sense RNA virus. Annual Review of Phytopathology, 59, 351–371. 10.1146/annurev-phyto-020620-113020 34077238

[mpp13179-bib-0028] Yao, M. , Zhang, T.Q. , Zhou, T. , Zhou, Y.J. , Zhou, X.P. & Tao, X.R. (2012) Repetitive prime‐and‐realign mechanism converts short capped RNA leaders into longer ones that may be more suitable for elongation during rice stripe virus transcription initiation. Journal of General Virology, 93, 194–202. 10.1099/vir.0.033902-0 21918010

