# Supplementary material for: The cap‐snatching frequency of a plant bunyavirus from nonsense mRNAs is low but is increased by silencing of UPF1 or SMG7
Source: Mol Plant Pathol. 2021 Dec 26;23(4):576–82. doi: 10.1111/mpp.13179 (PMC8916216; doi:10.1111/mpp.13179)
Supplement: Supplementary file 1 — FILE S1. Materials and methods; Table S1 Primer sequences [file MPP-23-576-s001.docx]

**Materials and methods**

**Plant materials and virus inoculation**

*Nicotiana benthamiana* were grown in a growth chamber at 25°C with a photoperiod of 16 h light/8 h dark. RSV was collected from Jiangsu and maintained in rice by transmission with *Laodelphax striatellus* Fallen. Crude extracts of RSV-infected rice were used to inoculate *N. benthamiana* as described previously (Zheng et al., 2015).

**Plasmid construction**

The plasmid pCHF_3_-C^12^ is a point mutant of a previously described plasmid named pCHF_3_-A^12^ (Lin et al., 2020). Similarly, the pCHF_3_-C^11^-PTC is a point mutant of pCHF_3_-A^11^-PTC, which itself is derived from a previously described plasmid named pCHF_3_-A^11^ by introducing a point mutation at codon 3 of the GFP coding region. The plasmids pHB-PHA-m and pHB-PHA-n were constructed using a recombination-based cloning strategy. The coding sequence of PHA-n was chemically synthesized from Takara Bio, China and PHA-m was created by introducing a point mutation at codon 79 of PHA-n. pTRV2-UPF1 and pTRV2-SMG7 was constructed according to a previous report of Kerényi et al. (2008). The primers used for the construction of the VIGS vectors are presented in table S1.1. All the plasmids were verified by sequencing before being transformed to *Agrobacterium tumefaciens* strain GV3101.

**Agroinfiltration**

The agroinfiltration used to present defined mRNAs to RSV was done as described previously (Lin et al., 2020). Briefly, *A. tumefaciens* carrying each plasmid were grown overnight in LB liquid medium supplemented with appreciate antibiotics. Agrobacterial cells were harvested and then resuspended with an agroinfiltration solution [10 mM 2-(N-morpholino) ethanesulfonic acid (MES), pH 5.6, 10 mM MgCl_2_ and 150 μM acetosyringone] at a final OD_600_ of 0.4. The suspensions carrying the plasmid of interest were mixed at a volume to volume ratio of 1:1 before being infiltrated to a fully expanded leaf of RSV-infected plants. The agroinfiltration used to silence *UPF1* or *SMG7* was done similarly except that cell suspensions with a final OD_600_ of 0.8 was used.

**qRT-PCR**

Standard qRT-PCR was used to determine the silencing efficiency of *UPF1*/*SMG7* and to examine the relative expression of PHA-m in UPF1/SMG7-silenced plants. In these experiments, total RNA was extracted from plant samples using TRIzol (Life technologies). cDNA was generated from 1 μg of the total RNA using oligo-d(T) as the primer after removing gDNA. qPCR was performed using real-star SYBR mixture (Genstar) according to manufacturer’s instructions. The primer pair PHA-qPCR-F/PHA-qPCR-R was used to quantify the expression of *PHA-*m/*PHA-*n. The primer pairs UPF1-qPCR-F/UPF1-qPCR-R or SMG7-qPCR-F/SMG7-qPCR-R were used to quantify *UPF1* and *SMG7*, respectively. The primer pair F-box-F/F-box-R was used to detect a *F-box* mRNA (Niben.v0.3.Ctg24993647), which was used as the internal reference of the qRT-PCR.

A strand-specific qRT-PCR was used to examine the accumulation of RSV vRNA3 or vcRNA3. This method was adopted from a report of Kawakami et al. (2011). Central to this method is the use of an oligo-tagged primer to reverse-transcribe the strand of interest with removing gDNA and the use of an primer corresponding to the tag in PCR. In our experiments, the oligo-tagged primer for vRNA3 (Segment3-vcRNAtag-RT) anneals to a region 3^’^ to *NP (*within the ORF of *NS3)*, so that the NP mRNA was not targeted. The oligo-tagged primer for vcRNA3 anneals to a region 3^’^ to NS3 to avoid the targeting of NS3 mRNA in reverse-transcription. The oligo-tagged cDNA was amplified with the primer pairs Segment3-vRNAtag-F/vRNAtag-qPCR-R and Segment3-vcRNAtag-F/vcRNAtag-qPCR-R in PCR examining the expression of vRNA3 and vcRNA3, respectively.

The primers used in the standard and strand-specific qRT-PCR were presented in Table S1.1.

**Northern blotting**

Northern blotting was used to detect the accumulation of RSV RNA3 in *UPF1*/*SMG7*- silenced and control plants. To do this, total RNA was extracted from 0.1 g of each plant with the hot phenol method. The total RNAs were separated on a 1% formaldehyde electrophoresis gel before being transferred to a nylon membrane (HybondN^+^) (GE Healthcare). Blots were hybridized with a biotin-labeled DNA probe (Themo scientifc). The probe was a PCR amplicon corresponding to a 507 bp fragment within the ORF of RSV *NP*. Labeled probes were detected by Chemiluminescent Nucleic Acid Detection Module Kit (Themo scientifc).

**Deep sequencing of RSV *NP***

Deep sequencing of RSV NP was performed as described previously (Liu et al., 2018; Lin et al., 2020). The total RNA was first treated with alkaline phosphatase (NEB). The dephosphorylated RNA was then treated with RppH (NEB), which de-caps mRNAs, leaving a monophosphate at their 5^’^ ends. After that, a RNA oligo (RNAlinker_8N) was ligated to the mRNAs with T4 RNA Ligase 1 (NEB, USA). The oligo-tagged RSV NP mRNA was reverse-transcribed with the primer RLM-RSV-NSvc3-outer-R. The cDNA was amplified with the primer pair Adaptor-PCR-F/RLM-RSV-NSvc3-inner-R (Table S1). The purified PCR amplicon around 250 nt in size were sent to Novogene for library construction and deep sequencing with a HiSeq 2500 platform.

**References**

Kawakami, E., Watanabe, T., Fujii, K., Goto, H., Watanabe, S., Noda, T., *et al*. (2011). Strand-specific real-time RT-PCR for distinguishing influenza vRNA, cRNA, and mRNA. *Journal of virological methods*, 173, 1-6.

Kerényi, Z., Mérai, Z., Hiripi, L., Benkovics, A., Gyula, P., Lacomme, C., *et al*. (2008). Inter-kingdom conservation of mechanism of nonsense-mediated mRNA decay. *EMBO Journal*, 27, 1585-1595.

Lin, W., Wu, R., Qiu, P., Jin, J., Yang, Y., Wang, J., *et al*. (2020). A convenient in vivo cap donor delivery system to investigate the cap snatching of plant bunyaviruses. *Virology*, 539, 114-120.

Liu, D., Shi, L., Han, C., Yu, J., Li, D., Zhang, Y. (2012). Validation of reference genes for gene expression studies in virus-infected Nicotiana benthamiana using quantitative real-time PCR. *PLoS one*, 7, e46451.

Liu, X., Jin, J., Qiu, P., Gao, F., Lin, W., Xie, G., *et al*. (2018). Rice Stripe Tenuivirus Has a Greater Tendency To Use the Prime-and-Realign Mechanism in Transcription of Genomic than in Transcription of Antigenomic Template RNAs. *Journal of virology*, 92, e01414-e01417.

Zheng, L., Du, Z., Lin, C., Mao, Q., Wu, K., Wu, J., *et al.* (2015). Rice stripe tenuivirus p2 may recruit or manipulate nucleolar functions through an interaction with fibrillarin to promote virus systemic movement. *Molecular plant pathology*, 16, 921–930.

Table S1 Primers sequences

| Name | Sequence (5^’^-3^’^) |
| --- | --- |
| UPF1-TRV2-F | agaaggcctccatggggatccAAGGTCCACCTGGTACGGG |
| UPF1-TRV2-R | cgtgagctcggtaccggatccGAGCAGCTTTCTTGCACATAATGAC |
| SMG7-TRV2-F | agaaggcctccatggggatccCCTCATCATCAGCTNGCAATACTG |
| SMG7-TRV2-R | cgtgagctcggtaccggatccACGAAAACCAAAACACCAGGCAAT |
| UPF1-qPCR-F | CCTTGAGCTTCGTGCCAGTC |
| UPF1-qPCR-R | CTTCGTGACCTAACAGGTGATGG |
| SMG7-qPCR-F | GAGGAACTCCGTGCACGCTT |
| SMG7-qPCR-R | CTCCTAGTGGCAGGCCATAC |
| GFP-qPCR-F | GGAGAGGGTGAAGGTGATGC |
| GFP-qPCR-R | CACGTATCCCTCAGGCATGG |
| PHA-qPCR-F | CCCTAGCCCTCTTCCTTGTG |
| PHA-qPCR-R | GCCCAGAGAGCTCAACGTGG |
| F-box-F | GTGCCCTCCCATGGACCTT |
| F-box-R | CGAGACCGCCCGAAGAACC |
| Segment3-vRNAtag-RT | *GGCCGTCATGGTGGCGAAT*GCGACTCTGATTGGCATACTGAAG |
| Segment3-vRNAtag-F | CCACCGAGGACACTATCCCATAC |
| Segment3-vcRNAtag-RT | *GCTAGCTTCAGCTAGGCATC*GCCCTTTACCGTTCCTAGTGTC |
| Segment3-vcRNAtag-F | GTGCACTAAGAGGTGGTTTATCAG |
| F-box-RT-R | CGTCAACCCATCTAACCCTGAG |
| vRNAtag-qPCR-R | GGCCGTCATGGTGGCGAAT |
| vcRNAtag-qPCR-R | GCTAGCTTCAGCTAGGCATC |
| pCHF3-GFP-PCR-F | GAGGATCCAAGGAGATATAACAATG |
| linker-GFP-PCR-R | GTAGTGACAAGTGTTGGCCATG |
| RNAlinker_8N | TCTACrArGrUrCrCrGrArCrGrArUrCrNrNrNrNrNrNrNrN |
| Adaptor-PCR-F | GTTCTACAGTCCGACGATC |
| RLM-RSV-NSvc3-outer-R | TTGTCACATCCTGTTAGTCCCAGG |
| RLM-RSV-NSvc3-inner-R | CAGCCGCCACTTTCACTTTCTTAG |
