# Supplementary material for: The cap‐snatching frequency of a plant bunyavirus from nonsense mRNAs is low but is increased by silencing of UPF1 or SMG7
Source: Mol Plant Pathol. 2021 Dec 26;23(4):576–82. doi: 10.1111/mpp.13179 (PMC8916216; doi:10.1111/mpp.13179)
Supplement: Supplementary file 2 — FILE S2. Table S2 Statistics for data sets obtained from wild‐type Nicotiana benthamiana co‐expressing GFP‐m and GFP‐n (the reciprocal experiment); Table S3 Statistics for data sets obtained from wild‐type N. benthamiana co‐expressing PHA‐m/PHA‐n and GFP; Table S4 Statistics for data sets obtained from UPF1‐silenced, SMG7‐silenced, or control N. benthamiana co‐expressing GFP‐m and GFP‐n; Table S5 Statistics for data sets obtained from UPF1‐silenced, SMG7‐silenced, or control N. benthamiana co‐expressing PHA‐m/PHA‐n and GFP [file MPP-23-576-s002.docx]

Table S2 Statistics for data sets obtained from wildtype *Nicotiana benthamiana* co-expressing GFP-m and GFP-n (the reciprocal experiment)

| Samples | total NP sequences | GFP-n-NP | GFP-m-NP |
| --- | --- | --- | --- |
| C^11^+ C^12^-PTC-NP-1 | 57,576 | 349 | 122 |
| C^11^+ C^12^-PTC-NP-2 | 72,146 | 228 | 121 |
| C^11^+ C^12^-PTC-NP-3 | 87,763 | 547 | 273 |

Table S3 Statistics for data sets obtained from wildtype *N. benthamiana* co-expressing PHA-m/PHA-n and GFP

| Samples | total NP sequences | PHA-n-NP | PHA-m-NP | GFP-NP |
| --- | --- | --- | --- | --- |
| PHA-n-NP-1 | 1,409,593 | 7,413 | - | 5,920 |
| PHA-n-NP-2 | 869,949 | 7,941 | - | 4,104 |
| PHA-n-NP-3 | 1,352,795 | 14,164 | - | 11,131 |
| PHA-m-NP-1 | 829,675 | - | 1,382 | 6,894 |
| PHA-m-NP-2 | 972,719 | - | 807 | 5,557 |
| PHA-m-NP-3 | 835,038 | - | 1,158 | 5,808 |

Table S4 Statistics for data sets obtained from *UPF1*-silenced, *SMG7*-silenced or control *N. benthamiana* co-expressing GFP-m and GFP-n

| Samples | total NP sequences | GFP-n-NP | GFP-m-NP |
| --- | --- | --- | --- |
| TRV-LUC-C^12^+ C^11^-PTC-NP-1 | 279,883 | 154 | 117 |
| TRV-LUC-C^12^+ C^11^-PTC-NP-2 | 494,650 | 184 | 120 |
| TRV-LUC-C^12^+ C^11^-PTC-NP-3 | 463,823 | 168 | 119 |
| TRV-UPF1-C^12^+ C^11^-PTC-NP-1 | 1,206,557 | 1,149 | 902 |
| TRV-UPF1-C^12^+ C^11^-PTC-NP-2 | 617,191 | 6,47 | 315 |
| TRV-UPF1-C^12^+ C^11^-PTC-NP-3 | 520,504 | 200 | 127 |
| TRV-SMG7-C^12^+ C^11^-PTC-NP-1 | 482,308 | 56 | 88 |
| TRV-SMG7-C^12^+ C^11^-PTC-NP-2 | 452,127 | 83 | 195 |
| TRV-SMG7-C^12^+ C^11^-PTC-NP-3 | 634,609 | 101 | 161 |

Table S5 Statistics for data sets obtained from *UPF1*-silenced, *SMG7*-silenced or control *N. benthamiana* co-expressing PHA-m/PHA-n and GFP

| Samples | total NP sequences | PHA-n-NP | PHA-m-NP | GFP-NP |
| --- | --- | --- | --- | --- |
| TRV-LUC-PHA-n-NP-1 | 779,806 | 2,481 | - | 1,460 |
| TRV-LUC-PHA-n-NP-2 | 929,776 | 4,213 | - | 3,633 |
| TRV-LUC-PHA-n-NP-3 | 339,751 | 900 | - | 1,119 |
| TRV-LUC-PHA-m-NP-1 | 927,485 | - | 399 | 1,960 |
| TRV-LUC-PHA-m-NP-2 | 562,196 | - | 194 | 1,516 |
| TRV-LUC-PHA-m-NP-3 | 631,622 | - | 335 | 2,079 |
| TRV-UPF1-PHA-n-NP-1 | 62,7129 | 2,155 | - | 1,425 |
| TRV-UPF1-PHA-n-NP-2 | 92,1657 | 1,504 | - | 887 |
| TRV-UPF1-PHA-n-NP-3 | 36,2948 | 501 | - | 410 |
| TRV-UPF1-PHA-m-NP-1 | 985,267 | - | 943 | 3563 |
| TRV-UPF1-PHA-m-NP-2 | 1,068,410 | - | 1,358 | 1409 |
| TRV-UPF1-PHA-m-NP-3 | 1, 001,074 | - | 833 | 1476 |
| TRV-SMG7-PHA-n-NP-1 | 581,327 | 1,954 | - | 1579 |
| TRV-SMG7-PHA-n-NP-2 | 1,095,164 | 3,527 | - | 3141 |
| TRV-SMG7-PHA-n-NP-3 | 882,497 | 3,756 | - | 2284 |
| TRV-SMG7-PHA-m-NP-1 | 721,112 | - | 172 | 969 |
| TRV-SMG7-PHA-m-NP-2 | 400,234 | - | 123 | 1,348 |
| TRV-SMG7-PHA-m-NP-3 | 1,242,849 | - | 628 | 2,859 |
